# The State of the Art of Diagnostic Multiparty Eye Tracking in Synchronous Computer-Mediated Collaboration

**DOI:** 10.16910/jemr.16.2.4

**Published:** 2023-06-19

**Authors:** Tom Frank Reuscher, Peyman Toreini, Alexander Maedche

**Affiliations:** Karlsruhe Institute of Technology, Germany

**Keywords:** eye movement, eye tracking, gaze, attention, computer-mediated communication, social interaction, collaboration

## Abstract

In recent years, innovative multiparty eye tracking setups have been introduced to synchronously capture eye movements of multiple individuals engaged in computer-mediated collaboration. Despite its great potential for studying cognitive processes within groups, the method was primarily used as an interactive tool to enable and evaluate shared gaze visualizations in remote interaction. We conducted a systematic literature review to provide a comprehensive overview of what to consider when using multiparty eye tracking as a diagnostic method in experiments and how to process the collected data to compute and analyze group-level metrics. By synthesizing our findings in an integrative conceptual framework, we identified fundamental requirements for a meaningful implementation. In addition, we derived several implications for future research, as multiparty eye tracking was mainly used to study the correlation between joint attention and task performance in dyadic interaction. We found multidimensional recurrence quantification analysis, a novel method to quantify group-level dynamics in physiological data, to be a promising procedure for addressing some of the highlighted research gaps. In particular, the computation method enables scholars to investigate more complex cognitive processes within larger groups, as it scales up to multiple data streams.

## Introduction

In synchronous collaboration, defined as working together in real-time to achieve a common goal, our visual perception plays an important role in understanding the characteristics and constraints of a task and the social context in which it is embedded (13; 15). Furthermore, gaze behavior has a crucial communicative function (11). For instance, “where one looks, how long, and when” signals engagement, supports rapport building, and regulates turn-taking in natural social interaction (14; 37).

During the last decade, novel eye tracking technologies have been introduced and leveraged to capture interdependent visual behavior between individuals by implementing two eye trackers synchronously (27). This dual eye tracking methodology has been used to study attentional processes in social interaction. The interdependent states of visual attention between individuals are defined as social gaze including joint attention (at least two individuals look at the same object), mutual gaze (two individuals look at each other) and gaze aversion (one individual looks at another who looks away; 11; 26). Previous research on social gaze has revealed fundamental characteristics of mutual gaze and provided interesting insights into its role in face-to-face communication (see 14). In the context of computer-mediated communication, dual eye tracking has mainly been used as a tool to exchange gaze information between remotely interacting individuals (see, e.g., 21). D’Angelo and Schneider (10) systematically reviewed the existing literature on the interactive use of eye tracking for shared gaze visualizations and discussed potential applications as well future research avenues.

Due to the global pandemic and worldwide confinements, virtual meetings have been established as an efficient alternative to face-to-face communication. Therefore, understanding the characteristics of social interaction in remote settings is more important than ever. Dual eye tracking is a promising methodology to investigate interdependent cognitive processes between individuals in computer-mediated collaboration. For instance, Jermann et al. (17) observed that individuals adapt their gaze behavior depending on the expertise of their partner in a cooperative version of Tetris. Moreover, the authors were able to predict a dyad’s combined expertise level (Novices, Experts, Novice/Expert) by synthesizing action- and gaze-based features in two machine learning recognition models. Further studies in this field focused on the quantification of gaze-based group variables, especially joint attention, and their correlation with various collaborative processes (7; 9; 16). As collaboration in virtual meetings is not limited to dyadic interaction, scholars extended the basic dual eye tracking setup by including additional eye trackers to study larger group sizes (see 38). Accordingly, in this article, we refer to the method of synchronously tracking the eye movements of at least two individuals as multiparty eye tracking.

Recently, a comprehensive overview of multiparty eye tracking setups for the study of social interaction has been provided by Valtakari et al. (34). However, the authors limited their review to studies investigating gaze in face-to-face communication. To the best of our knowledge, no literature review on the diagnostic use of multiparty eye tracking in remote interaction exists. By providing a holistic overview of the findings, limitations, as well as future opportunities, we aim to contribute to a better understanding of the promising methodology and its application to study interdependent cognitive processes in computer-mediated collaboration. As part of the present article, we developed an integrative conceptual framework to synthesize what needs to be considered when synchronously capturing eye movements of multiple individuals engaged in remote interaction and how to use the data to compute and analyze group-level eye movement metrics, such as social gaze.

## Methods

In order to address the outlined research gap, we conducted a systematic literature review following the guidelines by Kitchenham and Charters (19). Accordingly, the review process was divided into three stages – plan, conduct and report. In the planning phase, an efficient search strategy was developed by creating a specified search string and defining criteria to be followed when selecting relevant literature. In the conducting phase, the search strategy was executed on appropriate research data bases. Based on the extracted data, the conceptual framework of state-of-the-art diagnostic multiparty eye tracking in computer-mediated collaboration was developed. The framework was later used to report findings in detail.

### Search Strategy

The development of the search strategy started with an initial exploration on Google Scholar by applying the following terms: “eye tracking” AND “collaboration”. After reviewing a sample of relevant papers and according keywords, we defined a first version of the search string and specified it by several iterations. The final search string consisted of three parts (see Table 1).

**Table 1.** First, second, and third part of the final search string.

(1)	(“eye tracking” OR “eye movements” OR gaze)
(2)	AND (dual OR dyad* OR triad* OR multiparty)
(3)	AND (collaborat* OR “problem solving”)

The first part covered the most frequently used terms for indicating eye tracking experiments. Furthermore, we included common keywords relating to a multiparty eye tracking setup as the second part. The third part limited the search to the context of collaboration. Our initial investigation highlighted that several studies referred to collaboration as joint problem solving. Therefore, we also included this term. Furthermore, we decided not to add another part to the search string that limits the scope to studies explicitly referring to a remote setting. However, computer-mediated communication was defined as an inclusion criterion. To collect all relevant literature, the next step was to compare various electronic databases by checking whether the previously identified sample of highly relevant papers could be found in them. As a result, a combination of Scopus, Web of Science, ACM Digital Library and EBSCOhost was selected to be appropriate for executing the search. We chose them to cover different research domains as it is an interdisciplinary topic.

### Selection Criteria

In a further step, we defined the inclusion and exclusion criteria to be followed when reviewing and selecting literature for data extraction (see Table 2).

**Table 2.** Four criteria for selecting appropriate studies to answer the research question.

Inclusion	Synchronous Eye Tracking
	Experimental Collaboration Task
	Remote Interaction
Exclusion	Evaluation of Shared Gaze Visualization

According to the scope of this article, only eye tracking studies were selected. Due to the specific focus on collaboration, the first criterion was further specified to the synchronous tracking of at least two participants’ eye movements. Furthermore, the experimental task had to be characterized by a common objective and thus had to include some type of collaborative activity as well as a measurable outcome variable, such as team performance. To address the specific context of computer-mediated collaboration, only studies that featured remote interaction by providing individual and visually separated systems were included. Finally, studies primarily concerned with the evaluation of shared gaze visualizations based on multiparty eye tracking approaches were excluded, since related findings are already covered by D’Angelo & Schneider (10) and do not contribute to the goal of this article.

### Data Extraction

In the conducting phase, we executed the search strategy by applying the final search string to the selected databases. First, title and abstract of the identified literature were scanned following the defined selection criteria. Next, this procedure was repeated reviewing full texts. Finally, in an attempt to capture the entirety of relevant literature, a forward and backward search was performed on Google Scholar by checking the references of remaining studies and reviewing all articles that cited them. By carefully analyzing the final sample of papers, relevant aspects for answering the research question were defined iteratively. The extracted data was tabulated accordingly to report findings in a structured way (see Table 3). Finally, the conceptual framework was created by synthesizing the identified aspects.

**Table 3.**
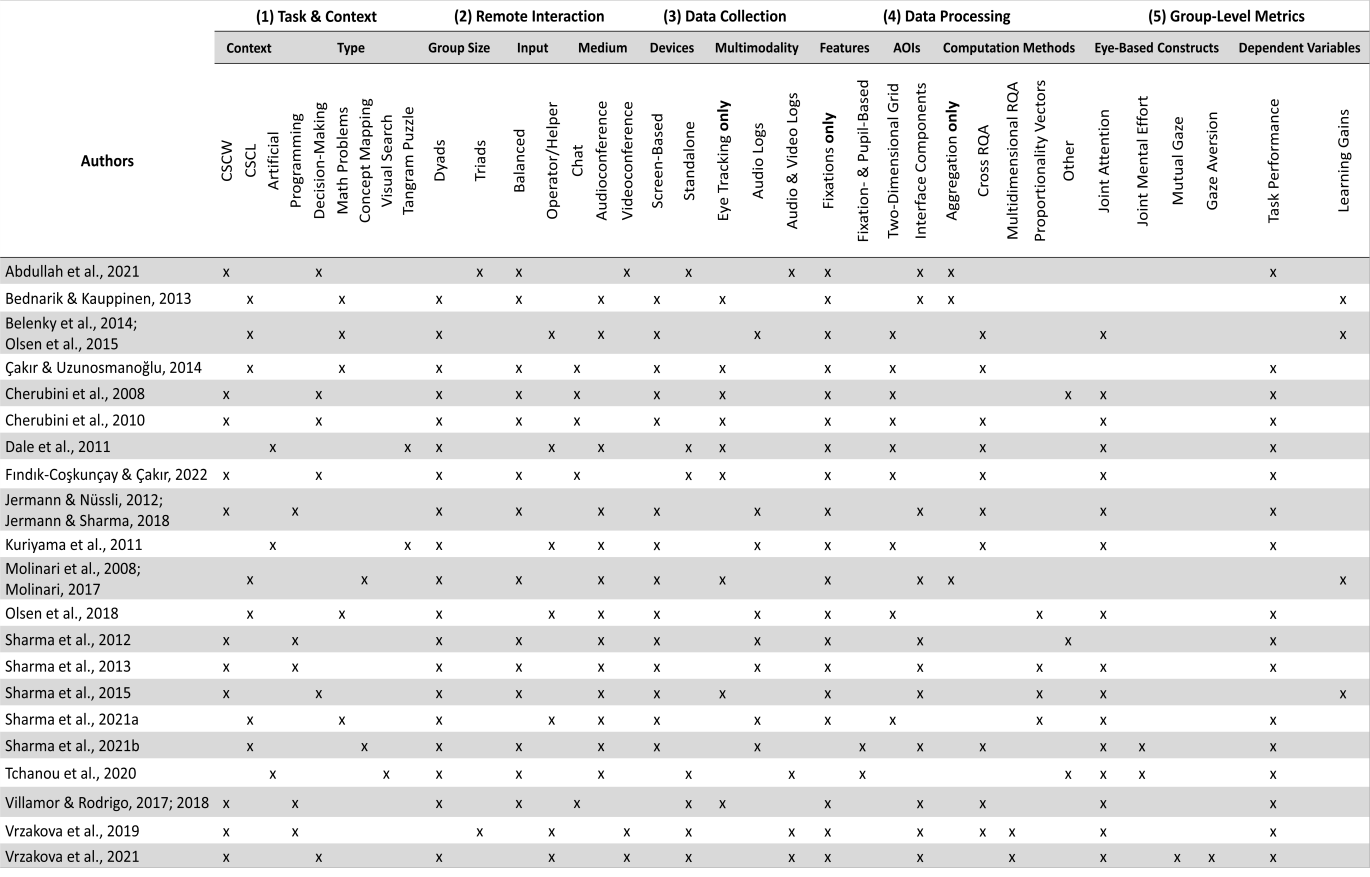
Extracted data tabulated by identified relevant aspects. Studies that matched in all relevant aspects are grouped in one row.

## Results

In this section, we first present the results of the review process in order to illustrate how the final sample of relevant literature was acquired. In a subsequent step, the conceptual framework is introduced and described in detail by referring to the identified dimensions.

### Review Process

**Figure 1.**
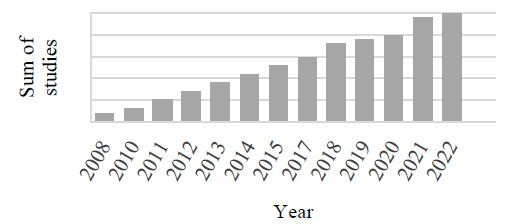
Cumulated number of studies by year of publication.

The execution of the search strategy resulted in 1665 initial hits (ACM Digital Library: 1354 hits; Scopus: 189 hits; Web of Science: 84 hits; EbscoHost: 38 hits). 1529 irrelevant studies were excluded by scanning title and abstract. Next, another 114 were excluded by reviewing full texts. The forward and backward search performed with the remaining 22 studies resulted in another 3 hits. Thus, the final sample of literature consisted of 25 relevant studies (see Table 4; Figure 1).

**Table 4.** Consecutive steps of the executed search strategy with number of remaining and excluded studies.

Title/Abstract	1665 (-1529)
Full text	136 (-114)
Forward/Backward	22 (+3)
Final sample	25

### Conceptual Framework

**Figure 2.**
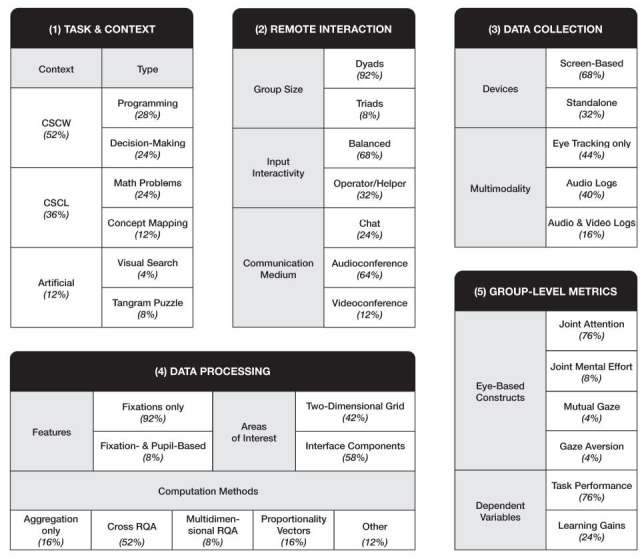
Integrative conceptual framework.

The integrative conceptual framework was developed by analyzing the extracted data and categorizing the identified aspects following a bottom-up procedure (see Figure 2). Relevant aspects to consider when using multiparty eye tracking diagnostically are summarized in the first three dimensions. Specifically, the first dimension – Task & Context – comprises detailed information on the experimental task and collaborative activity performed by participants. The second dimension – Remote Interaction – includes crucial characteristics of the interaction context in which the task is embedded. The eye tracking devices used in examined studies and the modality of additionally collected communication signals are condensed in the third dimension – Data Collection. The fourth and fifth dimensions include information on how the synchronized eye tracking data is processed to calculate group-level metrics. In particular, the fourth dimension summarizes important aspects related to the specific procedures – Data Processing, whereas the operationalized eye-based multiparty constructs and investigated dependent variables are synthesized in the fifth dimension – Group-Level Metrics.

Dimension 1: Task & Context. Based on the particular activities performed by collaborators, most tasks could be assigned to either a computer-supported cooperative work- (CSCW; 52%) or computer-supported collaborative learning-related context (CSCL; 36%). The remaining tasks were labelled as artificial (12%).

CSCW related tasks included software design activities referred to as collaborative programming (28%) and cooperative decision-making tasks characterized by the coordination on multiple attributes (24%). For instance, errors in a presented software code had to be discovered and marked in a programming task (36). One of the decision-making tasks required two participants to discuss and agree on eight key features related to a car deal in order to maximize their collective profit (37). Experimental tasks associated with CSCL included the collaborative solving of mathematical problems using educational collaboration tools (24%) and the joint creation of concept maps based on previously processed learning materials (12%). The three tasks described as artificial included a psychological change blindness task (4%; Tchanou et al., 2020) and two tangram puzzle games (8%; 9; 20).

Dimension 2: Remote Interaction. Most studies investigated behavior in dyadic interaction (92%). Only two studies tracked eye movements of up to three participants simultaneously (8%). Furthermore, the ability to interact with the tasks interface differed between the eye-tracked participants in eight studies, as they were assigned either an operator or helper role (32%). In the other studies, all participants could interact with the interface equally using manual input devices (68%). Furthermore, the richness of the social interaction was determined by the provided communication medium, ranging from simple chats (24%) to audio- (64%) and mixed-media videoconferences (12%).

Dimension 3: Data Collection. According to the focus on computer-mediated collaboration, only desktop-mounted eye trackers were used in the studies. These were either screen-based devices integrated into monitors (68%) or standalone devices set up next to the screen (32%). Moreover, most studies not only captured participants’ eye movements (44%), but also other communication signals, such as speech and body language by recording audio (40%) and video logs (16%).

Dimension 4: Data Processing. The computation methods used in the examined studies were based on either the position or duration of gaze points (92%). Two studies performed additional calculations with pupil size data (8%; 32; 33). In order to compute group-level metrics, data processing followed a similar procedure based on the synchronized eye movement data of individual participants. First, the interface was divided into smaller segments by applying a two-dimensional grid (42%) or defining specific components of the user interface (58%) as areas of interest (AOIs). Next, individual gaze metrics, such as the duration of fixations to the AOIs, were calculated (16). In a subsequent step, the participants’ individual metrics were used to perform the following group-level calculations: Cross recurrence quantification analysis (CRQA; 52%), multidimensional recurrence quantification analysis (MdRQA; 8%), proportionality vector analysis (16%) and other alternative approaches (12%), such as fixation clustering (6). In the remaining studies, computations were limited to simple aggregations, such as the sample’s proportional distribution of gaze to distinct AOIs (16%; 1; 3; 23; 22).

Dimension 5: Group-Level Metrics. Most studies performed the group-level computation methods to quantify joint attention (76%). Two studies additionally operationalized the extent of joint mental effort (8%) by calculating a group’s cognitive load based on synchronized pupil size data (32; 33). Vrzakova et al. (37), on the other hand, also analyzed social gaze dynamics, such as mutual gaze (4%) and gaze aversion (4%). Examined studies investigated correlations between these group-level eye movement metrics and either learning gains (24%) or task-specific performance variables (76%).

## Discussion

By conducting the systematic literature review, we identified relevant aspects that need to be considered when synchronously capturing and analyzing eye movements of multiple participants engaged in computer-mediated collaboration. In this section, methodological differences in the usage of diagnostic multiparty eye tracking as well as implications for future research avenues are critically discussed along the conceptual framework.

Despite adhering to the guidelines by Kitchenham and Charters (19), the outlined review process is subject to some limitations. First, the continuous development of the search string by iteratively adding relevant keywords might have ultimately resulted in excluding relevant studies. Another limitation might stem from the explicit choice on selection criteria and appropriate databases for executing the search. Furthermore, the relevance of extracted data was subjectively assessed, which might have influenced the conceptualization of the proposed framework.

### Synchronized Collaboration

In order to investigate eye movements and gaze patterns of collaborating participants within a corresponding visual space, user interfaces were shared in real time, contained a synchronized area, or were duplicated within dyads (22; 30; 37). Considering these differences, the degree of coupling differed between tasks. Interfaces updated in real-time, such as in the programming task introduced by Vrzakova et al. (38), enabled participants to jointly attend to changes on screen. Unsynchronized content, on the other hand, served more as an aid for verbal coordination on multiple aspects (1; 6). Although coordination is necessary for successful collaboration, these tasks do not allow for anticipating other participants’ visually recognizable actions. Thus, simple coordination games characterized by static interfaces might not be sufficient for capturing the underlying aspects of interactive visual behavior in computer-mediated collaboration.

Furthermore, the ecological validity of the activities performed differed greatly between tasks. Gaze patterns identified in a synchronized visual search task, such as the change blindness task introduced by Tchanou et al. (33), might not be comparable to those observed in more naturalistic activities, because eye movements reflect attentional processes that are specific to the particular task performed. However, artificial tasks might help to answer fundamental questions on the perceptual nature of visual behavior in computer-mediated collaboration, whereas studies using more naturalistic tasks could provide design guidelines for CSCW and CSCL related applications.

### Computer-Mediated Interaction

When investigating visual behavior in virtual settings, certain properties of the remote interaction context need to be considered. First, the number of involved participants determines the interaction’s complexity (38). For instance, joint attention (i.e., at least two individuals look at the same object) is deterministic in dyadic interaction (AB), but can take place in four variants between participants involved in triadic interaction (AB, AC, BC, ABC; 26). Thus, depending on the investigated gaze construct, group size might systematically affect the complexity of visual patterns related to the computer-mediated collaboration per se. Despite the fact that many CSCW and CSCL related activities exceed a number of two collaborators, only two studies investigated eye movements in triadic interaction, leaving a gap in research that needs to be addressed in the future (see 1; 38).

Moreover, participants were assigned either an operator or helper role in nine studies. This creates an imbalance between collaborators as only one is able to actively manipulate the tasks interface. As a result, the validity of comparisons within a dyad is questionable as attentional processes related to the eye movements differed. For example, Belenky et al. (4) introduced a mathematical task that enabled one participant to enter answers whereas the other could only support verbally. However, in most of the examined studies, participants were equipped with manual input devices enabling them to equally contribute to the solution of the task.

Furthermore, the richness of the computer-mediated communication differed between studies as the degree of synchronization between participants and the presence of verbal and nonverbal cues was determined by the communication medium featured (2). Chats, for example, restricted communication to text-based messaging, whereas mixed-media videoconferences enabled speech as well as the transmission of facial expressions, gestures, and body-language in real-time. In addition, dynamic components of the interface, such as chat boxes or videos, might naturally attract a participant’s attention causing systematic differences in gaze when compared to audioconferences that do not include any interactive area for communication. Therefore, findings regarding the visual behavior of participants cannot be generalized, because specific layout characteristics of the communication medium need to be taken into account. As computer-mediated collaboration in education and workforce is primarily realized by videoconferencing, this communication medium should be featured in future studies.

### Multiparty Eye Tracking Setup

Eye movements of the participants have to be tracked synchronously to capture interdependent dynamics of visual behavior. Although this requirement was already addressed by the inclusion criteria of this review, we identified considerable differences in the practical implementation of multiparty eye tracking. Recent studies used standalone desktop-mounted eye trackers instead of screen-based systems. This increases the area of application and makes it possible to integrate eye tracking into more naturalistic settings. Furthermore, some studies used chin rests in order to prevent head movements and limit a participant’s field of view to the screen (16; 29). Although this is a standard procedure to improve data quality, it might affect the generalizability of results, because it does not reflect natural behavior in computer-mediated collaboration. Thus, scholars should aim for ecological validity by using unconstrained state-of-the-art desktop-mounted eye tracking devices (see 36).

In addition, most studies also collected audio and video logs in order to investigate the relationship between gaze and other communication signals. Predominantly, the association between eye movements and speech was analyzed as referring expressions can be precursors of joint visual attention (9; 20; 25; 30). Since eye movements are naturally linked to other communication signals, multimodal data collection approaches should be considered in future research on computer-mediated collaboration.

### Computation Methods

As previously mentioned, studies followed a similar processing procedure that included the division of the interface into smaller segments, the calculation of each participant’s individual metrics and finally the specific computation method to quantify group-level eye movement metrics.

**Figure 3.**
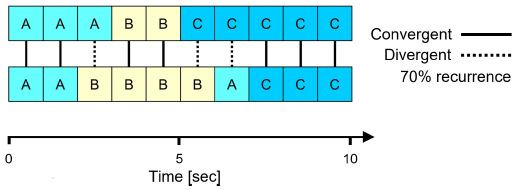
Exemplary time series of two participants with assigned AOIs (A, B, or C) per one-second intervals. The time series show a 70% recurrence as gaze converged in seven out of ten intervals.

Cross Recurrence Quantification Analysis (CRQA). The majority of studies used CRQA to identify the degree of convergence between two participants’ gaze locations over time. In order to identify recurrent states between two temporal streams of eye movement data, the individual time series of each participant’s fixations are initially cut into equal intervals (e.g., one-second slices). Next, each interval is assigned the AOI that contained the majority of gaze points during the selected duration. Finally, the recurrence rate between two participants is quantified by calculating the proportion of converging AOIs along the segmented time series (see Figure 3).

**Figure 4.**
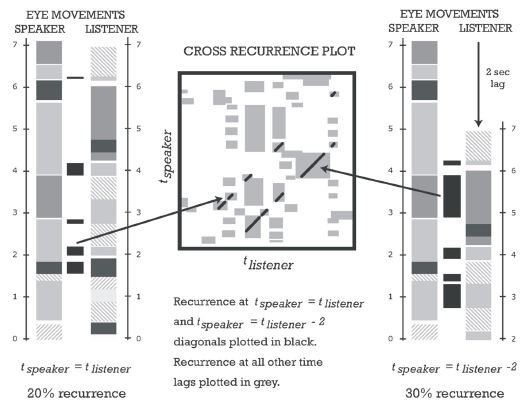
Illustration from Richardson & Dale (27): Time series of two participants (speaker, listener) at temporal synchrony (left) and a lag of two seconds (right).

The procedure can be repeated for any time lag between both data streams by shifting the segmented series of one participant in time. This is an essential aspect, as gaze is typically not visually transmitted in computer-mediated interaction and thus, might not converge in real time, but after a short period of time (9). For instance, Richardson and Dale (27), who were the first to perform CRQA on eye movement data, examined the delay of attentional coupling between speakers and listeners and found the highest recurrence rate at a lag of approximately two seconds (see Figure 4). CRQA can be performed with any type of data that contains dynamic states in temporal order, such as variations in pupil size over time (32). However, in examined studies it was almost exclusively performed with gaze coordinates to infer spatial convergence.

**Figure 5.**
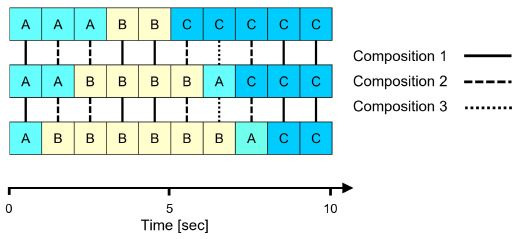
Exemplary time series of three participants with assigned AOIs (A, B, or C) per one-second intervals. Shown are episodes in which three participants look at the same AOI (composition 1; 50% recurrence), only two participants look at the same AOI (composition 2; 40% recurrence), and three participants divide their gaze between the AOIs (composition 3; 10% recurrence).

Multidimensional Recurrence Quantification Analysis (MdRQA). Recently, Vrzakova et al. (38) performed MdRQA, a novel extension of CRQA, to quantify dynamic states of visual attention between multiple participants. Instead of measuring the degree of convergent states between two time series, MdRQA measures the extent of recurring state compositions between numerous temporal data streams (see Figure 5).

Thus, MdRQA can be used to investigate more complex processes between individuals by operationalizing constructs of interest based on certain AOI compositions (see, e.g., 37). The avoidance of eye contact during social interaction, for instance, is not characterized by convergence, but systematic divergence between two participant’s gaze positions and therefore could not be computed using CRQA. Despite their usefulness for analyzing temporal dynamics between multiple data streams, both recurrence analysis methods have been found to be subject to confounding effects limiting the validity of group comparisons. For a detailed discussion of associated problems and possible solutions, we recommend the work of Coco and Dale (8) regarding CRQA and Wallot et al. (39) for MdRQA.

**Figure 6.**
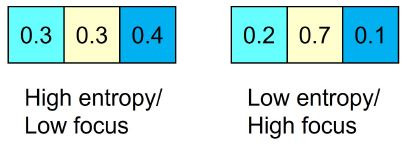
Adapted from Sharma et al. (31): Individual focus size based on high (left) and low entropy (right) of gaze across different AOIs (A, B, or C).

Proportionality Vector Analysis (PVA). Sharma et al. (30) developed PVA as an alternative fixation-based method to measure the degree of gaze similarity between two participants. The procedure is based on the analysis of two-dimensional vectors that reflect the proportion of time each participant spent looking at defined AOIs within a short period of time (i.e., A: 20%; B: 40%; C: 40%). Instead of measuring the rate of gaze convergence between two time series at a particular time lag, the extent to which both participants’ gaze dispersed between the AOIs is quantified. PVA includes two procedural steps (31). First, the Shannon entropy of each participant’s vector series is calculated to quantify whether they focused on a few or many different AOIs within a given time span (see Figure 6).

**Figure 7.**
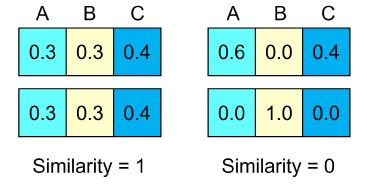
Adapted from Sharma et al. (31): Similarity of gaze distribution between AOIs (A, B, or C) for perfectly matching (left) and completely different entropy values (right) of two participants.

In a subsequent step, the similarity between two participants’ gaze patterns is computed by calculating either the scalar product or the reverse function of the proportionality vectors correlation matrix (25; 28). As a result, a similarity value of one indicated a consistent pattern of gaze distribution between two participants, whereas lower values indicated less similar patterns (see Figure 7).

Compared to recurrence analysis, the computation method is easier to perform as it requires fewer procedural steps. However, the conceptual differences between gaze convergence and gaze similarity have to be considered when investigating collaborative patterns in computer-mediated collaboration. In contrast to CRQA, the exact order of fixation locations along a time series is not taken into account. Thus, spatio-temporal dynamics of collaborative gaze could only be investigated by conducting recurrence analysis (35; 36).

**Figure 8.**
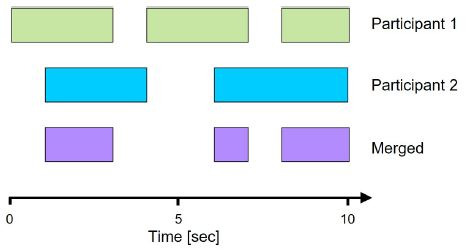
Adapted from Sharma et al. (29): Exemplary time series depicting two participants’ prolonged sequences of stable gaze patterns (green, blue) as well as their convergent episodes merged together (purple).

Alternative Approaches. Sharma et al. (29) introduced a segmentation method to distinguish between convergent and divergent episodes during dyadic interaction. This was accomplished by initially splitting each participant’s time series into equal slices. In a further step, consecutive slices with the same amount of fixated AOIs were accumulated and segmented as prolonged sequences of stable patterns. Next, the segmented series of both participants were temporally aligned in order to identify and merge intersections into a new time series of convergent episodes (see Figure 8).

The segmentation method should be considered when comparing episodes of visual behavior characterized by different degrees of coupling between participants. However, the proposed operationalization of convergence based on the range of fixated AOIs might be misleading, because the extent to which gaze spatially matched between participants was not taken into account (31). The definition is similar to the construct of gaze dispersion that was later introduced by the authors as part of the analysis of proportionality vectors (see 30).

**Figure 9.**
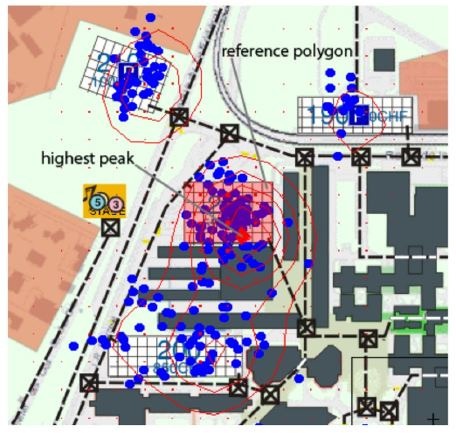
Illustration from Cherubini et al. (6): Shown are the participants’ gaze positions (blue) and plots of the contour function used to compute the gaze density peaks (red).

Furthermore, Cherubini et al. (6) developed a clustering method to locate spatial zones of interest within the interface based on the position of single fixations. To accomplish that, the interface was divided by a two-dimensional grid in order to compute a gaze density matrix on the basis of fixations within each cell. After smoothening the data using a Gaussian filter, gaze density peaks were located by applying a contour function to the gaze density matrix. Finally, the mean distance between participants’ density peaks was taken to quantify the degree of visual coupling (see Figure 9).

### Eye-Based Constructs

**Figure 10.**
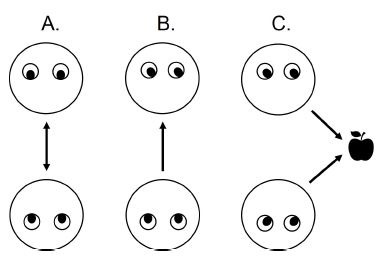
Adapted from Pfeiffer et al. (26): Mutual gaze (A; two individuals look at each other), gaze aversion (B; one individual looks at another who looks away), and joint attention (C; at least two individuals look at the same object).

Overall, four distinct eye-based multiparty constructs were operationalized: joint attention, mutual gaze, gaze aversion, and joint mental effort. As calculated from spatial gaze data (e.g., fixations), the first three constructs represent interdependent attentional processes between collaborating individuals. Together, they are known as the core dynamics of social gaze (see Figure 10; 11; 26).

Rather than examining the extent of visual coupling based on spatial gaze data, two studies measured the similarity in cognitive load between team members, defined as joint mental effort. Cognitive load describes the extent of cognitive resources expended while processing a task (33). In contrast to joint attention, mutual gaze, and gaze aversion, joint mental effort is calculated from pupil-based data, since cognitive load and pupil size are positively correlated (32).

Joint Attention. Examined studies almost exclusively quantified joint attention defined as visual coupling in terms of either gaze convergence, similarity or overlap (5; 31). In general, positive correlations between the extent of joint attention and learning gains as well as task-related performance variables were observed (16; 28, 33). For instance, Belenky et al. (4) found that pairs of students exhibited higher learning gains when maintaining a high level of joint attention throughout the collaborative use of an intelligent tutoring system. Moreover, Villamor and Rodrigo (36) observed a significantly higher group performance when a dyad’s gaze converged more frequently. In addition, they were able to show that the higher performing participants tend to lead the collaborative process as their fixation locations preceded the other participants’ ones in time. In contrast, Cherubini et al. (6) did not find evidence for a relationship between task performance and joint attention when computing the alternative fixation clustering method. Interestingly, a positive association was observed performing CRQA with the exact same data set (7).

Mutual Gaze & Gaze Aversion. The two social gaze dynamics that occur directly between individuals (i.e., at least one looks at another; see Figure 10) were investigated by only one of the examined studies (37). Whereas mutual gaze has previously been found to be a key factor for efficient communication and coordination, the level of gaze aversion has shown to be indicative of competitive behavior (see 37). Since participants cannot see each other’s gaze in computer-mediated interaction, mutual gaze is operationalized as the dynamic pattern, when both participants simultaneously look at the AOI associated with each other’s video in mixed-media conferences. Accordingly, gaze aversion is defined as the state, when one participant looks at another participant’s video, while this one is looking somewhere else on the screen. Consistent with previous findings, Vrzakova et al. (37) observed a negative correlation between gaze aversion and team performance. Mutual gaze occurred less frequently in computer-mediated communication when compared to similar face-to-face studies. However, no significant correlation between mutual gaze and any of the dependent variables was found.

Joint Mental Effort. Sharma et al. (32) performed CRQA to determine the extent of convergence in pupil size between participants and thus compute the eye-based multiparty construct of joint mental effort. Specifically, a high recurrence rate indicated that participants worked closely together as their individual effort levels converged over time. The authors found that joint mental effort was significantly higher in high performing dyads. In addition, a positive correlation between joint attention and joint mental effort as computed by recurrence analysis was observed. Thus, a complementary approach of analyzing fixation- and pupil-based data should be considered in future studies, because joint mental effort might be another valid indicator for successful collaboration.

### Conclusion

Several implications for future research on visual behavior in computer-mediated collaboration were derived from the results of this systematic literature review. Specifically, we identified fundamental requirements related to the data acquisition. In order to make valid comparisons between individual participants, any confounds of the experimental task that might elicit systematic differences in patterns of their visual attention need to be ruled out a priori. This includes, for example, the exact synchronization of the visual space between collaborating participants and the assignment of equal operating roles. In order to achieve the aforementioned and to enhance the overall generalizability of findings, future studies should consider controlled, artificial tasks instead of highly specific activities, such as pair programming. Moreover, audio- and videoconferencing tools are recommended to feature the computer-mediated communication, as writing and reading chat messages naturally attracts visual attention. Finally, a replication of findings with at least three participants is necessary as research was mainly limited to computer-mediated collaboration in dyadic interaction. MdRQA is a promising computation method to address some of the identified research gaps. Since it scales up to more than two synchronized data streams, spatio-temporal dynamics of visual behavior can be investigated in larger groups. In addition, more complex multiparty eye-based constructs, such as mutual gaze and gaze aversion, can be studied as the method not only measures basic alignment, but the extent to which systematic state compositions recur over time.

### Ethics and Conflict of Interest

The authors declare that the contents of the article are in agreement with the ethics described in http://biblio.unibe.ch/portale/elibrary/BOP/jemr/ethics.html and that there is no conflict of interest regarding the publication of this paper.

### Acknowledgements

Funded by the Deutsche Forschungsgemeinschaft (DFG, German Research Foundation) – GRK2739/1 – Project Nr. 447089431 – Research Training Group: KD²School – Designing Adaptive Systems for Economic Decisions.
